# Human Embalming Techniques With Organ Preservation: An Update and Literature Review

**DOI:** 10.7759/cureus.106287

**Published:** 2026-04-01

**Authors:** Edivaldo X Silva Júnior, Maria A Bettencourt-Pires, Livia C França Silva, Anna L Morais Silva, Vitoria C De Souza, Valentina B Vassilenko

**Affiliations:** 1 Laboratory for Instrumentation, Biomedical Engineering and Radiation Physics (LIBPHYS), NOVA School of Science and Technology, NOVA University of Lisbon, Lisbon, PRT; 2 Human Anatomy Teaching and Research Laboratory (LABEPAH), University of Pernambuco, Petrolina, BRA; 3 Department of Anatomy, NOVA Medical School (NMS), NOVA University of Lisbon, Lisbon, PRT

**Keywords:** cadaver, embalming, hemodynamics, histology, instillation, perfusion, preservation

## Abstract

The present review focuses on the importance of modern embalming techniques for human cadaveric preservation, comparing recent innovations with other methods and their historical evolution. Early approaches to human embalming for medical purposes presented significant limitations. During the past century, formaldehyde became widely adopted due to its low cost and high preservation efficiency. However, its use has been progressively restricted in modern laboratories because of the considerable health risks to professionals and its limitations in maintaining the organic properties of tissues. Consequently, more recent alternative preservation methods have been increasingly explored and considered within contemporary anatomical practice. We performed a thorough literature review in search of the best alternatives to formaldehyde for human cadaveric conservation with histological preservation. Eight of the recent studies under analysis propose ameliorations of older techniques, while others enhance the performance of original innovations in embalming techniques. The results show that none of these innovations demonstrates complete efficiency to substitute formaldehyde. It is, therefore, urgent to develop new alternative embalming techniques that are able to preserve the histologic integrity of human cadaveric material while preserving the biosafety of professional handlers.

## Introduction and background

The use of human cadaveric material for anatomical demonstrations is fundamental for the success of health courses. This implies the preparation of embalmed human corpses [1-3] through impregnation of chemical fixators to substitute necrotic odors, to disinfect, and to retard tissular degradation [4].

The earliest reports of embalming techniques come from Ancient Egypt, around 4300-3100 BCE, for religious purposes [5]. Such procedures could be reproduced and ameliorated to modern times, but with different purposes. Throughout history, one can trace reports on different approaches to human embalming. Ruysch was the Dutch medical anatomist of the 17th century, who first used the venous path for the instillation of embalming fluids. We found, however, no reports on what compounds he used. Not long after, in the following century, the Scottish surgeons William and John Hunter refined the technique by perfusing the femoral artery with perfumes in combination with mercurial compounds. The problem was the short duration of embalming [6]. Later, in the 19th century, Gannal’s method was developed in France for the embalming of human cadaveric parts and segments through the injection of a preservative solution into the carotid artery, composed of arsenic acid, zinc sulfate, and distilled water. This technique was subsequently tested in Brazil by José Tavano, but it yielded unsatisfactory results. One of the reasons for the failure of this technique resides in climatic differences between Europe and South America. Nevertheless, Gannal was also severely criticized by his compatriot and contemporary, Sucquet, who only used zinc chloride, after what he learned from Ruysch [7].

In the late 19th and early 20th centuries, embalming techniques greatly improved, particularly with respect to the longevity of cadaveric material. Formaldehyde was introduced by Coleman and Kogan in the UK [8] or Garret Jr. in the US [9], and Thiel, in Germany, developed innovative alternatives to formaldehyde [10]. In Portugal, in 2013, Goyri-O’Neill et al. developed a compound of aliphatic alcohols, such as ethylene glycol derivatives, to be instilled through larger arteries, such as the femoral or carotid arteries [11]. This method was considered the best alternative by Júnior et al., Brenner, and Balta et al. [4,12,13]. Nowadays, in Brazil, one of the most widely used embalming methods still uses formaldehyde instillation. This procedure implies the injection of the compound in a liquid state to impregnate the whole cadaver or several isolated cadaveric parts.

Formaldehyde is a low-cost, colorless, flammable, and odorless gas that retards the degradation time of cadaveric decomposition. Modernly, several limitations and restrictions to this technique are well established, although it may still be in use in some countries worldwide. Apart from its low cost, good disinfectant/sanitization, and good preserving properties, this product should, nevertheless, be considered as inadequate for anatomy teaching or research, due to the severity of risks associated with its use. Moreover, it demonstrates poor efficiency in preserving the freshness characteristics of cadaveric material, such as color, viscosity, or texture. The stiffness of the preserved cadaveric material compromises the possibility of its use in surgical training, in addition to its classification as a carcinogenic substance by the International Agency for Research on Cancer [14]. These concerns have stimulated the search for alternative preservation techniques capable of improving biosafety conditions while maintaining the organoleptic and biomechanical properties of tissues [13,15]. Maintaining these characteristics is essential for contemporary anatomy education, as specimens that preserve flexibility, natural coloration, and reduced chemical odor provide a more realistic approximation of living tissues, enhancing spatial understanding, clinical reasoning, and procedural training among students [4]. Therefore, the development and evaluation of innovative embalming techniques represent an important technical, pedagogical, and occupational health advancement for anatomy laboratories and for the training of future health professionals.

In this context, this study aimed to review and critically analyze recent advances in human embalming techniques reported in the literature over the past five years, with particular emphasis on alternative preservation methods capable of reducing or replacing formaldehyde while preserving tissue freshness, flexibility, and the organoleptic characteristics of cadaveric specimens. Additionally, the review explores key aspects related to biosafety, applicability in anatomy education and surgical training, and the cost-effectiveness of these modern preservation approaches, considering the framework previously proposed by Goyri-O’Neill et al. [11].

## Review

Methodology

This study was designed as an integrative literature review conducted in accordance with the Preferred Reporting Items for Systematic Reviews and Meta-Analyses (PRISMA) guidelines [16], aiming to ensure methodological transparency, reproducibility, and rigor in the processes of identification, selection, and synthesis of the scientific evidence.

The integrative review approach, as described by Souza et al. [17], was adopted to allow the inclusion and critical appraisal of studies with diverse methodological designs, including experimental, theoretical, and empirical investigations. This approach enables a comprehensive understanding of the current state of knowledge regarding cadaver preservation techniques, particularly those proposed as alternatives to formaldehyde.

The literature review focused on the search for alternative methods to formaldehyde, to ameliorate the preservation of the histological quality of tissues from human corpses. Article selection was executed through a systematic use of the National Library of Medicine database (PubMed/MEDLINE), prioritizing scientific pertinence, methodological stringency, and evidentiary caliber of indexed studies within this preeminent, globally acknowledged repository of biomedical scholarship. PubMed was deliberately selected for its comprehensive compendium of peer-reviewed literature in medicine, health sciences, and allied biomedical domains, underpinned by exacting indexing protocols that safeguard the assimilation of publications evincing superior scholarly integrity. This strategic deployment facilitated the precise and reproducible curation of germane investigations consonant with the review's objectives, thereby obviating extraneous inclusions and fortifying the epistemological robustness of the bibliographic retrieval process. The literature search employed a meticulously curated array of keywords and controlled vocabulary terms, encompassing "instillation," "perfusion," "human cadaver," "corpse," "embalming," "organ," "tissue," and "histological findings."

**Figure 1:**
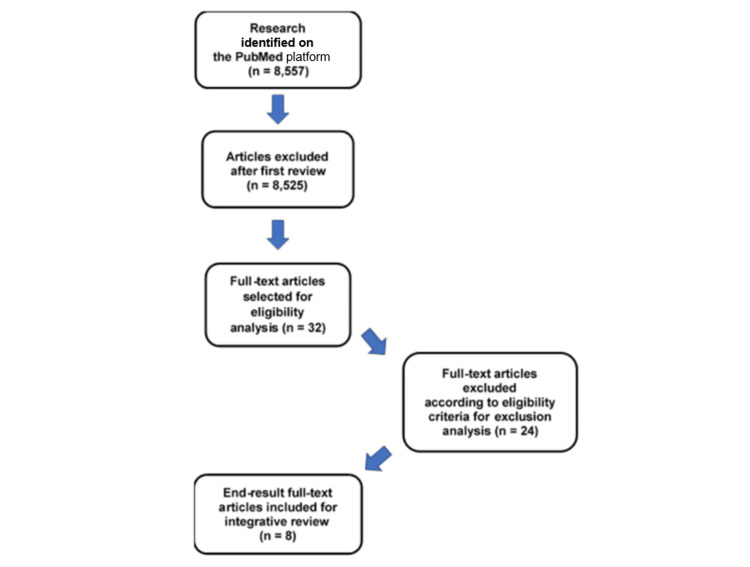
Flowchart of the process of selection of articles for integrative review

This review included peer-reviewed studies published between February 2018 and March 2023-a targeted five-year period to capture emerging innovations in human cadaver embalming techniques, prioritizing methods that minimize or eliminate formaldehyde use while preserving tissue integrity, histological quality, and organoleptic properties. Inclusion required (1) articles in English, Portuguese, or Spanish explicitly evaluating perfusion, instillation, or analogous fixation methods in human cadavers and (2) reporting of quantitative or qualitative outcomes on tissue preservation, structural integrity, or embalming efficacy specifically for anatomical education. Exclusion criteria were applied as follows: (1) studies utilizing animal models; (2) case reports or case series; (3) investigations based on surgical specimens, forensic samples, or pathological materials not intended for educational dissection; and (4) publications not directly relevant to the primary research objective of identifying formaldehyde alternatives (Figure 1).

The temporal restriction was justified by the rapid evolution of preservation technologies post-2018, enabling evaluation of innovative fixation methods that preserve organoleptic characteristics and histological fidelity. This approach facilitates a comprehensive synthesis of recent methodological advancements, extending beyond established formaldehyde-based protocols documented in prior literature.

The study selection followed the PRISMA flow structure, comprising four stages: identification, screening, eligibility, and inclusion. Initially, all records retrieved from the database were exported and screened for duplicates. Subsequently, titles and abstracts were independently reviewed by two researchers to assess their relevance according to the predefined eligibility criteria. Potentially eligible studies were then subjected to full-text analysis. Disagreements between reviewers were resolved through discussion and consensus, ensuring consistency and minimizing selection bias. When necessary, a third reviewer was consulted to reach a final decision (Table 1).

**Table 1:** Research eligibility criteria

Inclusion	Exclusion
Publication within 5 years	Animal cadaveric material
Corresponding to the research inquiry	Case studies
Titles referring to perfusion, organic preservation of human cadaveric material	Material collected from surgery, pathology, or forensic medicine

Data Extraction and Analytical Synthesis

Data from the included studies were systematically extracted using a standardized approach, considering variables such as preservation technique, type of fixation (e.g., perfusion or instillation), chemical composition, histological outcomes, tissue flexibility, color preservation, odor, and applicability in anatomical teaching and surgical training.

Given the heterogeneity of the included studies, a qualitative and thematic synthesis was performed. The findings were organized into three analytical categories: formaldehyde-based preservation techniques, vascular perfusion methods, and natural or alternative preservation processes. This categorization allowed a deeper analytical interpretation of the advantages, limitations, and applicability of each method, facilitating a critical comparison of their effectiveness, safety, and potential as substitutes for formaldehyde.

Methodological Considerations

Due to the limited number of studies specifically addressing embalming techniques for educational purposes, studies investigating broader post-mortem preservation methods were also included when they contributed to understanding the mechanisms of tissue conservation. This decision is consistent with the integrative review approach and allowed the incorporation of foundational knowledge essential for the development of innovative preservation strategies.

Results and discussion

The integrative review process resulted in the identification of 8,557 studies, of which 8,525 were excluded after title and abstract screening. Thirty-two articles were selected for full-text assessment, and eight met the established inclusion criteria. The final selection underwent independent and critical appraisal by reviewers, with consensus reached through structured discussion.

The included studies reflect a multidisciplinary scientific landscape, involving medical practitioners, biologists, archaeologists, physicists, epidemiologists, and a philosopher. This diversity reinforces the inherently interdisciplinary nature of cadaveric preservation research, spanning clinical, educational, forensic, and bioethical domains. However, it also contributes to methodological heterogeneity, which remains a critical limitation in the consolidation of standardized embalming protocols (Table 2).

**Table 2:** Main information on the selected journal articles

Journal	Title	Author/year	Type of research study
Autopsy Case Report	Formalin pre-fixation improves autopsy histology	Vazzano et al./2021	Experimental prospective study [18]
Folia Morphologica	The effect of prolonged formalin fixation on the staining characteristics of archival human brain tissue	Alrafiah and Alshali/2019	Experimental observational study [19]
Annals of Anatomy-Anatomischer Anzeiger	Recommendations of the working group of the Anatomische Gesellschaft on reduction of formaldehyde exposure in anatomical curricula and institutes	Waschke et al./2019	Non-experimental descriptive study [20]
Journal of Emergency Medicine	A novel expeditionary perfused cadaver model for trauma training in the out-of-hospital setting	Redman and Ross/2018	Development research [21]
Forensic Science International	Histomorphological assessment of isolated abdominal organs after targeted perfusion with the contrast agent Angiofil® in postmortem computed tomography angiography	Stumm et al./2020	Experimental and observational (mixed) study [22]
Journal of Forensic and Legal Medicine	Histology and Raman spectroscopy of limed human remains from the Rwandan Genocide	Schotsmans et al./2020	Observational descriptive study [23]
Bosnian Journal of Basic Medical Sciences	Histological observations on adipocere in human remains buried for 21 years at the Tomašica grave-site in Bosnia and Herzegovina	Salihbegović et al./2018	Retrospective observational study [24]
Spectrochimica Acta Part A: Molecular and Biomolecular Spectroscopy	Mummified embalmed head skin: SR-FTIR microspectroscopic exploration	Moissidou et al./2021	Observational descriptive study [25]

From a critical perspective, the heterogeneity observed across studies regarding preservation techniques, outcome measures, and analytical approaches highlights the absence of consensus in the field. Similar concerns have been raised in recent literature, emphasizing the need for standardization of embalming protocols to improve reproducibility and comparability across studies [12-15,17-26]. To facilitate analytical clarity, the selected studies were categorized into three major thematic groups: (1) formaldehyde-based techniques, (2) vascular perfusion techniques, and (3) natural preservation processes.

Formaldehyde-Based Techniques: Persistence Versus Toxicity

Despite the well-documented cytotoxic, mutagenic, and carcinogenic effects of formaldehyde, it remains the most widely employed fixative in anatomical sciences. In the present review, approximately 40% of the included studies relied on formalin-based preservation, underscoring its persistent dominance, primarily attributed to its proven efficacy in long-term tissue fixation, structural stability, and cost-effectiveness. This widespread use reflects not only its chemical efficiency in cross-linking proteins but also the logistical and economic constraints that still shape anatomical laboratory practices globally.

The findings of Vazzano et al. [18] provide an important nuance to this paradigm, demonstrating that short-term prefixation protocols may enhance histological quality while minimizing excessive tissue hardening and morphological distortion. This suggests that controlled exposure strategies can partially attenuate the deleterious effects traditionally associated with formaldehyde. Similarly, Alrafiah and Alshali [19] reaffirm the relevance of formaldehyde in maintaining specimen durability over extended periods, particularly in large-scale educational collections, where long-term preservation remains a critical requirement.

However, these advantages must be rigorously balanced against substantial occupational and environmental risks. Waschke et al. [20] and Pal et al. [27] highlight the cumulative toxicity of formaldehyde exposure, particularly in poorly ventilated laboratory settings, where chronic inhalation may lead to respiratory dysfunction, mucosal irritation, and increased cancer risk. These concerns are further reinforced by its classification as a Group 1 carcinogen by the International Agency for Research on Cancer, establishing unequivocal evidence of its carcinogenicity in humans. Such findings elevate the discussion beyond technical considerations, situating formaldehyde use within a broader framework of occupational health, bioethics, and institutional responsibility.

In response to these challenges, recent literature increasingly advocates for the reduction or complete elimination of formaldehyde in anatomical practice. Brenner [12], Kaliappan et al. [26], and Pal et al. [27] emphasize a paradigm shift toward low-toxicity or formaldehyde-free preservation methods, driven not only by biosafety imperatives but also by pedagogical demands. Contemporary anatomical education increasingly values tissue pliability, color fidelity, and near-physiological consistency-features that are often compromised by conventional formalin fixation.

Critically, the continued predominance of formaldehyde appears to be less a reflection of scientific superiority and more an outcome of entrenched practices, infrastructure limitations, and economic accessibility. This inertia highlights a significant translational gap between emerging scientific evidence and its implementation in educational and research environments. Therefore, there is a growing ethical, pedagogical, and occupational imperative to transition toward safer and more functionally adequate preservation techniques.

Ultimately, while formaldehyde remains an effective and accessible solution, its long-term sustainability in anatomical sciences is increasingly questionable. Advancing toward alternative methods is not merely a technological evolution, but a necessary shift aligned with contemporary standards of biosafety, educational quality, and respect for both human donors and laboratory professionals.

Vascular Perfusion Techniques: Functional Realism and Surgical Fidelity

Vascular perfusion techniques represent a major conceptual and technical advancement in cadaver preservation, particularly when the objective extends beyond static anatomical study to include surgical training, procedural simulation, and functional anatomical understanding. Unlike traditional immersion-based fixation, perfusion methods enable a more homogeneous distribution of preservative agents through the vascular network, thereby preserving not only macroscopic structures but also the integrity of microvascular architecture and tissue biomechanics.

Redman and Ross [21] demonstrated that saline perfusion of previously frozen cadavers maintains tissue pliability and structural coherence, offering a model that closely approximates in vivo mechanical behavior. This is particularly relevant for surgical simulation, where tactile feedback and tissue responsiveness are critical components of skill acquisition. These findings are consistent with the growing adoption of “soft-embalming” approaches, notably the Thiel method, which has been widely recognized for its superior preservation of flexibility, color fidelity, and joint mobility [26]. Such characteristics significantly enhance the translational value of cadaver-based training, bridging the gap between anatomical education and clinical practice.

Expanding the scope of perfusion-based techniques, Stumm et al. [22], although not primarily focused on embalming for educational purposes, introduced Angiofil-based vascular perfusion to optimize vascular visualization using advanced imaging modalities. This approach underscores an important paradigm shift toward integrative methodologies that combine anatomical preservation with diagnostic imaging technologies such as computed tomography (CT) and micro-CT. These hybrid models enable high-resolution anatomical mapping and three-dimensional reconstruction, offering unprecedented opportunities for both research and teaching.

Recent literature further consolidates the relevance of perfusion-based embalming in contemporary anatomical sciences. Júnior et al. [4] demonstrated that such techniques significantly enhance surgical training outcomes by preserving essential tissue properties, including elasticity, color, and anatomical relationships, which are often compromised in formalin-fixed specimens. Complementarily, Hammer [28] highlighted the effectiveness of the Thiel method in minimally invasive surgical training and radiological correlation, reinforcing its role as a gold standard for high-fidelity anatomical simulation. Together, these findings support the notion that perfusion-based preservation is not merely an alternative technique but a transformative approach in anatomical pedagogy.

Nevertheless, despite their clear advantages, perfusion techniques present notable limitations that restrict their widespread adoption. These methods often require specialized infrastructure, technical expertise, and higher operational costs, including the acquisition of specific reagents and controlled laboratory environments. Consequently, their implementation remains largely concentrated in well-resourced institutions, creating disparities in access to high-quality anatomical training. This scenario highlights a crucial challenge for the sector, which must keep the balance between technological advancement and global accessibility.

In this context, future research should focus not only on refining perfusion techniques but also on developing cost-effective and scalable adaptations that can be implemented in diverse educational settings. Bridging this gap is essential to ensure that the benefits of high-fidelity cadaver preservation extend beyond elite centers, contributing to a more equitable and effective anatomical education worldwide.

Natural Preservation Processes: Scientific Insight and Limitations

Studies on naturally preserved cadavers provide valuable insights into post-mortem biochemical processes, although their direct applicability to modern anatomical education remains limited. Schotsmans et al. [23] demonstrated that calcium carbonate application may delay macroscopic decomposition but fails to preserve microscopic tissue integrity. Similarly, Salihbegović et al. [24] identified adipocere formation as a key factor in natural preservation, influenced by environmental conditions such as humidity, soil composition, and anaerobic bacterial activity. Moissidou et al. [25] employed advanced spectroscopic techniques to analyze mummified tissues, demonstrating preservation at both structural and molecular levels; however, the absence of detailed characterization of the embalming compounds used limits the reproducibility and translational applicability of these findings.

Recent advances in biomolecular archaeology and forensic sciences have substantially expanded this field through the application of spectroscopic and molecular imaging techniques. In particular, Fourier transform infrared spectroscopy (FTIR) has demonstrated high analytical sensitivity in detecting structural and biochemical alterations in human tissues, enabling the identification of collagen degradation and mineral transformations associated with diagenetic processes [29]. Complementary techniques, such as laser-induced breakdown spectroscopy (LIBS), have further enhanced the molecular and elemental characterization of skeletal remains in complex burial environments, allowing for non-destructive and high-resolution analysis of degraded tissues [30]. These methodological advances reinforce the relevance of natural preservation studies, as they elucidate the physicochemical mechanisms governing tissue degradation and conservation, particularly the role of environmental variables-such as soil composition, humidity, and microbial activity-in modulating post-mortem pathways.

Importantly, this body of knowledge has direct implications for the development and refinement of cadaveric embalming techniques. Insights derived from natural preservation processes have informed the design of innovative preservation strategies aimed at maintaining tissue integrity, elasticity, and structural fidelity while reducing chemical toxicity. Contemporary evidence indicates that alternative approaches, including soft embalming and hybrid fixation techniques, benefit from these principles by more closely replicating in vivo tissue characteristics, thereby enhancing their applicability in anatomical teaching, experimental research, and surgical training environments [13]. In this context, cadaveric models preserved through advanced techniques have demonstrated superior performance in simulating real surgical conditions, contributing to improved procedural skill acquisition and deeper anatomical understanding.

Nevertheless, important limitations persist. Natural preservation processes are inherently variable and difficult to standardize, as they depend on complex and often uncontrollable environmental factors, which restrict their reproducibility and direct implementation in controlled laboratory settings. Furthermore, although biomolecular and spectroscopic analyses provide critical mechanistic insights, their routine incorporation into embalming protocols remains constrained by technical complexity and resource demands.

Collectively, the findings of this review delineate a field in transition. Conventional formaldehyde-based methodologies endure owing to their pragmatism and economic viability; however, they are increasingly challenged by safer and functionally superior alternatives. Formaldehyde-based techniques provide effective long-term preservation but at the expense of biosafety; perfusion methods offer enhanced anatomical fidelity, albeit with higher operational costs; and natural preservation strategies contribute substantial scientific insight, despite their limited practical applicability. Recent literature points toward a paradigm shift favoring hybrid and low-toxicity embalming techniques that integrate chemical innovation, imaging technologies, and pedagogical demands. Such approaches represent a promising avenue for advancing cadaveric preservation in a manner that aligns scientific rigor with the evolving needs of medical education, research, and surgical training.

## Conclusions

The present review critically analyzed recent advances in human embalming techniques over the past five years, with a particular focus on alternative preservation strategies capable of reducing or replacing formaldehyde while maintaining tissue freshness, flexibility, and organoleptic properties. The findings reveal a clear and consistent pattern that, despite technological progress and increasing scientific interest in alternative approaches, no method has yet demonstrated sufficient robustness, reproducibility, and scalability to fully replace formaldehyde-based fixation in anatomical sciences. Vascular perfusion techniques emerge as the most promising advancement, particularly in the context of surgical training and high-fidelity anatomical simulation. These methods offer superior preservation of biomechanical properties, tissue elasticity, and color fidelity, enabling a “near-life” experience that significantly enhances both undergraduate and postgraduate training. However, their implementation remains constrained by high costs, technical complexity, and infrastructural demands, limiting their accessibility on a global scale. Similarly, natural preservation processes and hybrid techniques contribute to a deeper understanding of post-mortem tissue dynamics but lack standardization and consistent applicability in educational settings.

Conversely, formaldehyde-based techniques persist as the predominant method, not due to scientific superiority, but rather because of their accessibility, low cost, and proven effectiveness in long-term preservation. This persistence reflects a critical gap between emerging scientific evidence and its practical implementation. Despite its classification as a human carcinogen and its well-established limitations in preserving life-like tissue characteristics, formaldehyde continues to dominate anatomical practice, highlighting an urgent need for a paradigm shift grounded in biosafety, ethical responsibility, and pedagogical innovation. Importantly, the absence of truly novel and fully effective embalming methods in the analyzed literature should not be interpreted as a limitation of this review, but rather as a significant scientific finding. It underscores that current research efforts have been predominantly directed toward refining and adapting existing techniques, rather than developing disruptive preservation technologies. Within this context, the framework proposed by Goyri-O’Neill remains one of the most comprehensive and effective models for cadaver preservation, a finding that is further supported by recent evidence, including our own investigations. Therefore, future research must move beyond incremental improvements and focus on the development of innovative, cost-effective, and scalable preservation strategies that reconcile three fundamental dimensions: (i) biosafety, through the reduction or elimination of toxic agents; (ii) functional realism, ensuring the preservation of tissue properties compatible with surgical training; and (iii) accessibility, enabling implementation across diverse educational and research environments. Ultimately, advancing cadaver preservation is not solely a technical challenge but a strategic imperative for the evolution of anatomical sciences. Bridging the gap between safety, realism, and feasibility will be essential to ensure that future health professionals are trained using anatomically accurate, ethically sound, and pedagogically effective models that closely reflect real clinical conditions.
